# Generalizability of motor modules across walking-based and in-place tasks – a distribution-based analysis on total knee replacement patients

**DOI:** 10.3389/fbioe.2025.1471582

**Published:** 2025-04-07

**Authors:** Mahziyar Darvishi, Sajjad Daroudi, Shahabedin Tavasoli, Ali Shafiezadeh, Farzam Farahmand

**Affiliations:** Mechanical Engineering Department, Sharif University of Technology, Tehran, Iran

**Keywords:** synergy analysis, non-negative matrix factorization, clustering, k-means, shared modules

## Abstract

**Introduction:** There are evidences that the nervous system produces motor tasks using a low-dimensional modular organization of muscle activations, known as motor modules. Previous studies have identified characteristic motor modules across similar tasks in healthy population. This study explored the generalizability of motor modules across two families of walking-based (level-walking, downhillwalking and stair-decent), in-place ascending (sit-to-stand, squat-to-stand), and in-place descending (stand-to-sit and stand-to-squat) motor tasks in a group of six individuals undergone total knee replacement (TKR) surgery.

**Methods:** Motor modules were extracted from the EMG data of CAMS-Knee dataset using non-negative matrix factorization technique. A distribution-based approach, employing three levels of k-means clustering, was then applied to find the shared and task-specific modules, and assess their representability among the whole task-trial data.

**Results and Discussion:** Results indicated a four- and a seven-subcluster arrangement of the shared and task-specific motor modules, depending upon the membership criteria. The first arrangement revealed motor modules which were shared across all tasks (min coverage index: 76%; modules’ distinctness range: 7.08–8.91) and the latter among tasks of the same family mainly, although there remained some interfamily shared modules (min coverage index: 81%; modules‘ distinctness range: 7.17–9.89). It was concluded that there are shared motor modules across walking-based and in-place tasks in TKR individuals, with their generalizability and representability depending upon the analysis method. This finding highlights the importance of the analysis method in identifying the shared motor modules, as the main building blocks of motor control.

## 1 Introduction

Human movements are complex due to two main factors. First, the issue of muscle redundancy, where multiple muscles can achieve the same movement, complicates how the central nervous system (CNS) selects which muscles to activate. Second, the degree of freedom (DOF) redundancy means the body has many joints and muscles that can move in multiple ways to accomplish the same task. It is often hypothesized that the central nervous system (CNS) produces tasks by implementing a low-dimensional modular organization of muscle activations, i.e., motor modules, to overcome these complexities (Ting and Macpherson, 2005; Clark et al., 2010; Darvishi et al., 2022a). Synergy analysis of the electromyography (EMG) envelopes can unveil the coordination between muscle activities and help to identify the motor modules as the building blocks of motor control, which are activated through time-dependent activation profiles to accomplish a motor task (Hayes et al., 2014; Shojaeefard et al., 2018; Mehryar et al., 2020). Recent studies have demonstrated that a similar analogy might be applied to the kinematic level, where the synergy analysis characterizes the coordination of the joint motions during complex movements and recognizes several kinematic modules or movement primitive as the building blocks (Darvishi et al., 2022b; Esmaeili et al., 2022; Tavasoli et al., 2023). Based on this concept, muscle synergies are thought to serve as the neural counterparts of the kinematic synergies, providing a structured mechanism for generating coordinated movements across various tasks. In fact, recent studies have shown that there is a one-to-one association between the motor modules and kinematic synergies, suggesting that each individual movement primitive might be implemented by the recruitment of a paired motor module (Esmaeili et al., 2022).

As a rational consequence of the synergy hypothesis, it might be assumed that different motor tasks are produced through appropriate activation of a limited number of common motor modules. Previous studies have shown that, in spite of the strikingly diverse mechanics and energetics, there are common muscle modules during walking, arm and leg cycling, and recumbent stepping (Zehr et al., 2007), treadmill walking and recumbent stepping (Stoloff et al., 2007), walking and running (Cappellini et al., 2006), walking and cycling (Barroso et al., 2014), walking and slipping (Nazifi et al., 2017), and walking and reactive balance (Allen et al., 2019). There are also reports of common motor modules when performing the same task with different conditions in humans and animals, e.g., walking at different speeds (Barroso et al., 2014).

The similarity of motor modules across different tasks has been often investigated in previous studies using scalar product (Barroso et al., 2014) and Pearson correlation coefficient (Routson et al., 2014), as well as fixing the modules based on one task and extracting the activation profiles of other tasks (Hug et al., 2011). An important limitation of all these studies, however, is examining the similarities between the averaged motor modules; this approach might be reasonably criticized for limiting the analysis to the trimmed averaged data which does not reflect the variations among population and trials. In order to examine if the subjects truly demonstrate shared motor modules across different tasks, the motor modules of different tasks should be compared in a distribution-based approach which weighs the whole task-trial data.

Moreover, only few studies have investigated the common synergies in the individuals with neuromusculoskeletal disorders and/or the tasks of dissimilar natures. Fox et al. (2013) studied the shared motor modules in children with incomplete spinal cord injury during a number of locomotion tasks and reported reduced generalizability compared to the control children. Also, Saito et al. (2021) investigated the shared motor modules in healthy subjects across some diverse motor tasks, i.e., locomotion, dynamic and static stability, and reported that 13 muscle synergies accounted for 24 tasks.

The objective of this study was to investigate the motor module generalizability across two families of walking-based and in-place motor tasks, in a group of total knee replacement (TKR) individuals with similar ages. In particular, we employed a distribution-based approach to examine the whole task-trail data for shared motor modules.

Understanding the nature and consistency of shared motor modules between different activities is vital for advancing our fundamental knowledge of how the central nervous system organizes and adapts motor commands. Demonstrating these common modules can provide insights into the capacity of both healthy and those with musculoskeletal issues—to transfer motor skills across tasks, thereby informing more effective motor learning and rehabilitation strategies.

Given that total knee replacement is a commonly performed surgery for end-stage knee osteoarthritis, understanding whether TKR individuals utilize similar neuromuscular control strategies (i.e., motor modules) across different tasks is crucial for developing targeted rehabilitation protocols. This research addresses an important gap in existing literature by assessing the consistency of these modules in a clinical population, and the findings have the potential to inform individualized rehabilitation strategies that leverage or adapt these shared motor control elements for improved functional outcomes.

## 2 Materials and methods

### 2.1 Data and processing

The muscle activity data used in this study were taken from the CAMS-Knee dataset (Taylor et al., 2017; CAMS-Knee, 2019) containing the EMG, kinematic and kinetic data from six subjects (5 male, 1 female, aged 68 ± 5 years, mass 88 ± 12 kg, height 173 ± 4 cm) who had undergone TKR with a cemented INNEX knee implant (FIXUC, Zimmer, Switzerland). The data was captured while the subjects performed multiple trials of free level walking, stair descent, downhill walking, Squat, and Sit-Stand-Sit. The surface EMG signals were acquired from 16 muscles in both legs (eight predominant muscles of each leg), including rectus femoris (RF), vastus medialis (VM), vastus lateralis (VL), tibialis anterior (TA), medial hamstrings (HM), lateral hamstrings (HL), medial gastrocnemius (GM), and lateral gastrocnemius (GL), with a sampling frequency of 2,000 Hz.

The EMG data of muscles were analyzed for seven tasks, categorized into two families of walking-based and in-place. The walking-based tasks included level walking (WLV), downhill walking (WDH), and stair descent (WSD), which were all considered to be consisted of standard cycles from heel strike to heel strike of the instrumented leg. The in-place activities included two ascending tasks, i.e., squat to stand (ASQ) and sit to stand (AST), and two descending tasks, i.e., stand to squat (DSQ) and stand to sit (DST). Each motion task required at least five valid trials to be conducted. All valid trials for each participant and each task were included in the analysis. A trial was considered valid for the walking-based tasks if the knee was within the field of view of the image intensifier during both the stance and swing phases, and if the force plates were struck accurately. For further details on the task explanation, please consult the provided resource (CAMS-Knee, 2019). The motion data (marker data) from the CAMS-knee dataset were visualized using the OpenSim software (V. 4.3) environment. For walking-based tasks, the heel markers were used to determine the timing of heel strikes. The period between the first heel strike of one limb and the subsequent heel strike of the same limb was defined as one cycle of walking-based tasks. For the in-place tasks, the sacrum marker was tracked; specifically, the time from when the sacrum was stationary to moving downward and then back to stationary was considered a descending cycle (DSQ and DST), while the time from stationary to moving upward was considered an ascending cycle (ASQ and AST).

The EMG data was pre-processed in MATLAB R2020. The EMG data was high-pass filtered (40 HZ, zero lag, fifth-order Butterworth), demeaned, full-wave rectified, low-pass filtered (4 HZ, zero lag, fourth-order Butterworth) (Allen and Neptune, 2012), and down-sampled by taking means using 10 msec time binning (Chvatal et al., 2011). For each trial of each task, the EMG data of each muscle was then normalized to its maximum value during that trial (Cheung et al., 2005). Finally, to enable better comparison between the results, the EMG data of each trial was resampled to form an 8 × 101 matrix.

### 2.2 Synergy analysis

For each trial, the muscle modules were extracted from the processed EMG envelope of that trial using Non-Negative Matrix-Factorization (NNMF) method in MATLAB R2020 (Ting and Macpherson, 2005). NNMF’s nonnegativity condition for the activation of basis vectors is particularly useful in identifying physiologically meaningful synergies, as it prevents outputs containing negative activation of the muscles. This feature makes NNMF an effective tool for analyzing muscle modules, ensuring that the extracted synergies reflect realistic muscle activations (Lambert-Shirzad and Van der Loos, 2017). The Variability Accounted For (VAF) measure was used to assess the reconstruction error, which represents the discrepancy between the original experimental EMG signals and those reconstructed from the extracted motor modules. The VAF quantifies how well the identified muscle synergies can explain the variance in the recorded EMG signals. VAF was calculated using the following formula:

$$VAF=1-\frac{{\sum \left({EMG}_{\exp}-{EMG}_{rec}\right)}^{2}}{\sum {\left({EMG}_{\exp}\right)}^{2}}$$

where EMG_exp_ represents the experimental EMG data, and EMG_rec_ represents the reconstructed EMG data. A higher VAF value indicates a better reconstruction and a lower reconstruction error.

The number of modules increased from one, and the optimal muscle modules and activation profiles were found using alternative least square method (Haghpanah et al., 2017) in MATLAB R2020. In order to ensure that the optimization is global, the procedure was repeated 500 times with random initial values (Esmaeili et al., 2022). For each trial, the reconstruction accuracy was assumed to be acceptable, if the VAF of all muscles (VAF_global_) was higher than 95%, and that of each muscle (VAF_local_) higher than 85% (Esmaeili et al., 2022).

For each task, the Most Frequent Number (MFN) of modules which met the VAF criteria across all trials was determined. The synergy analysis was then executed again on all task-trial data, with the MFN assumed as the number of modules.

### 2.3 Clustering and correlation analysis

This study employed a distribution-based approach to ensure that the entire set of motor modules across trials was considered in the generalizability analysis. Instead of averaging motor modules within a task, all task-trial modules were pooled, and their distributions were analyzed using a multi-level clustering process implemented in MATLAB R2020. First, for each task, the characteristic motor modules were found by an intra-task clustering process. The muscle modules of all task-trial data were clustered using k-means procedure, with the number of clusters set to the MFN of the task. Modules indicating Pearson correlations coefficient (*r*) less than 0.576 (corresponding to *p* = 0.05 for eight muscles) with the mean of the cluster were drawn out (Chvatal and Ting, 2013). Moreover, if two modules of a trial were in the same cluster, the one with lower correlation was removed. The centroids of the clusters were assumed to represent the characteristic muscle modules of the task.

In the next level, the characteristic motor modules were clustered in an inter-task clustering process, to find the shared characteristic modules across the tasks. The membership value was the same as in the previous step (*r* > 0.576), but the number of clusters was not pre-set. The clustering procedure started with the largest MFN across the tasks, assumed as the number of clusters. If the characteristic modules of the tasks were all assigned to clusters, the procedure was terminated; otherwise, it was repeated with adding a single unit to the number of clusters.

Finally, two steps of intra-cluster clustering process were performed to identify the representative motor modules and examine and improve their representability for the whole task-trial data. Initially, for each cluster of the previous step, the motor modules of all trials assigned to that cluster were pooled. Then in the first step, the pooled data were clustered by k-means, with the number of clusters set to one and applying a membership value of *r* > 0.576. The centroid of each cluster was considered as the representative of the common motor module, and its representability was assessed by the coverage index (CI), i.e., the number of cluster members over that of the pooled data. The second intra-clustering process was performed to identify common motor modules with improved representability among the task-trial data. The pooled data of each cluster was clustered again by k-means to identify subclusters of task-trial modules with similar structures. The number of subclusters started from one, and members showing low correlations (*r* < 0.576) with the centroid were eliminated. If 90% of the pooled data was assigned to the subclusters, the procedure was terminated; otherwise, a single unit was added to the number of subclusters, and the process was repeated. The centroids of the subclusters were considered as the representatives of the common motor modules.

After identifying the representative motor modules through the clustering process, the activation profiles for each task were calculated by averaging the activations of all the trials of different tasks assigned to a given cluster/subcluster. Specifically, for each representative motor module, the activation time series of all task-trial data categorized in that cluster/subcluster were extracted and averaged across trials to obtain the characteristic activation pattern of that module.

To visualize the clustering results, t-distributed Stochastic Neighbor Embedding (t-SNE) was applied in MATLAB R2020 (Laurens van der Maaten, 2008). Each task-trial’s m-dimensional motor module was projected into two-dimensional space. The consistency of the representative shared modules was quantified using the radius of the circle that encompassed 95% of the cluster points, *R*_*95*_, and their distinctness using the means of the distances between the *R*_*95*_ centers, *d* (Sawers et al., 2015).

### 2.4 Statistical analyses

To compare the distribution for the number of muscle synergies for trials of each of seven tasks (WLV, WDH, WSD, AST, DST, DSQ, and ASQ), a one-way analysis of variance (ANOVA) with *post hoc* multiple comparisons is used. Also, each of the LW, DHW, SD, AST, ASQ, DST, and DSQ motor module muscles are compared with the average (pooled) motor modules in terms of muscle activity through the exploitation of the two-sided t-test. All significance level for the statistical analyses is set to 0.05, and all results are reported as mean ± 1 standard deviation.

## 3 Results

**FIGURE 1:**
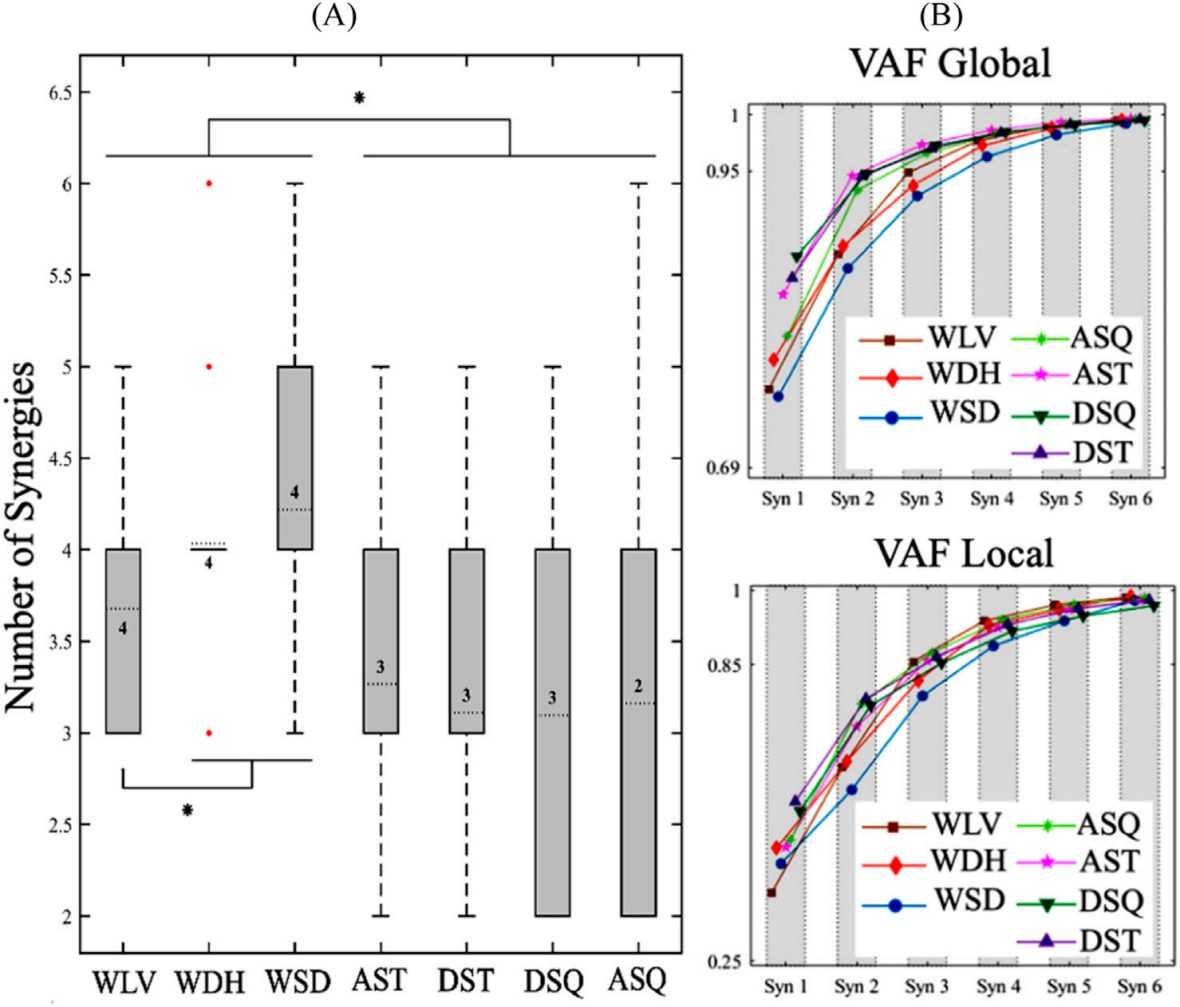
**(A)** Box plots and ranges of the number of muscle synergies for trials of each task. The numbers inside the boxes indicate the most frequent numbers (MFNs) of motor modules, and the red dots represent the outlier data for the WDH. Also, The * sign indicates a significant difference (p < 0.05). **(B)** The average VAF results for different numbers of muscle synergies of each task. WLV: level walking; WDH: downhill walking; WSD: stair descent, ASQ: squat to stand; AST: sit to stand; DSQ: stand to squat; DST: stand to sit.

The box plots and ranges of the number of muscle synergies for the trials of each task (Figure 1A) indicated relatively consistent results for the walking-based tasks, i.e., WLV, WDH, and WSD, as well as the in-place ascending tasks, i.e., ASQ and AST. In particular, for the WDH, all trials except for three, exhibited the same number of modules. The in-place descending tasks, i.e., DSQ and DST, demonstrated more variability in the number of modules among different trials. Statistical comparison of the number of synergies of different tasks indicated significantly larger numbers of modules for all walking-based tasks compared with all in-place tasks (*p* < 0.05). Also, among the walking-based tasks, the WSD and WDH had larger numbers of synergies than the WLV (*p* < 0.05). No significant difference was found between the number of muscle synergies of in-place tasks, nor between those of the WSD and the WDH. Based on the global and local VAF results (Figure 1B), the MFNs of different tasks were obtained as 4 for all walking-based tasks, 3 for all in-place tasks except for ASQ, and 2 for ASQ.

**FIGURE 2:**
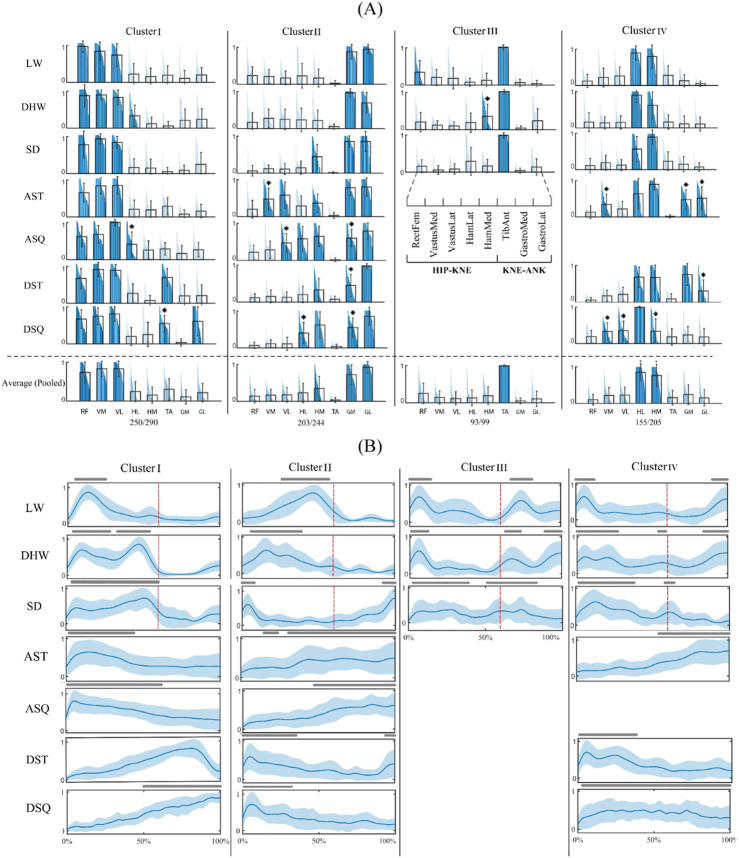
Four-cluster arrangement of shared motor modules. **(A)** Characteristic motor modules of different tasks (first seven rows) and the representative motor modules of each cluster and their coverage rate across all task-trial data (last row). The main contributing muscles into each module (relative weights larger than 0.3) are colored and * sign indicates a significantly different muscle weight from that of the representative module (p < 0.05). **(B)** Activation profiles of the muscle modules. The lines above each profile indicate activation levels above 50% of the maximum and red lines separate the stance and swing phases of the gait cycle. WLV: level walking; WDH: downhill walking; WSD: stair descent, ASQ: squat to stand; AST: sit to stand; DSQ: stand to squat; DST: stand to sit.

Clustering of the characteristic motor modules of the tasks resulted in a four-cluster arrangement of shared modules (Figure 2A). In this study, we regarded the main contributing muscles within each module as those with a relative weight exceeding 0.3. The representative modules of cluster I and cluster II were common among all tasks (CI: 86% and 83%, respectively). The representative module of cluster III included the TA as the main contributor and was shared by the walking-based tasks only (CI: 94%). Finally, the representative module of cluster IV contained the hamstring group as the main muscles and was common across all tasks, except for the ASQ. This module had the lowest representability (CI: 76%), mainly due to the contributions of muscles other than the hamstring group in the in-place tasks (*p* < 0.05).

The activation profiles of the modules of different tasks (Figure 2B) show how the shared motor modules reconstructed the muscle activation patterns of different tasks. In this study, a module was deemed active if its activation level surpassed 50% of the maximum. For instance, the module of cluster I was activated in the mid stance of WLV, mid stance to terminal stance of the WDH and WSD, from the beginning to the middle of the in-place ascending tasks, and from the middle to the end of in-place descending tasks.

**FIGURE 3:**
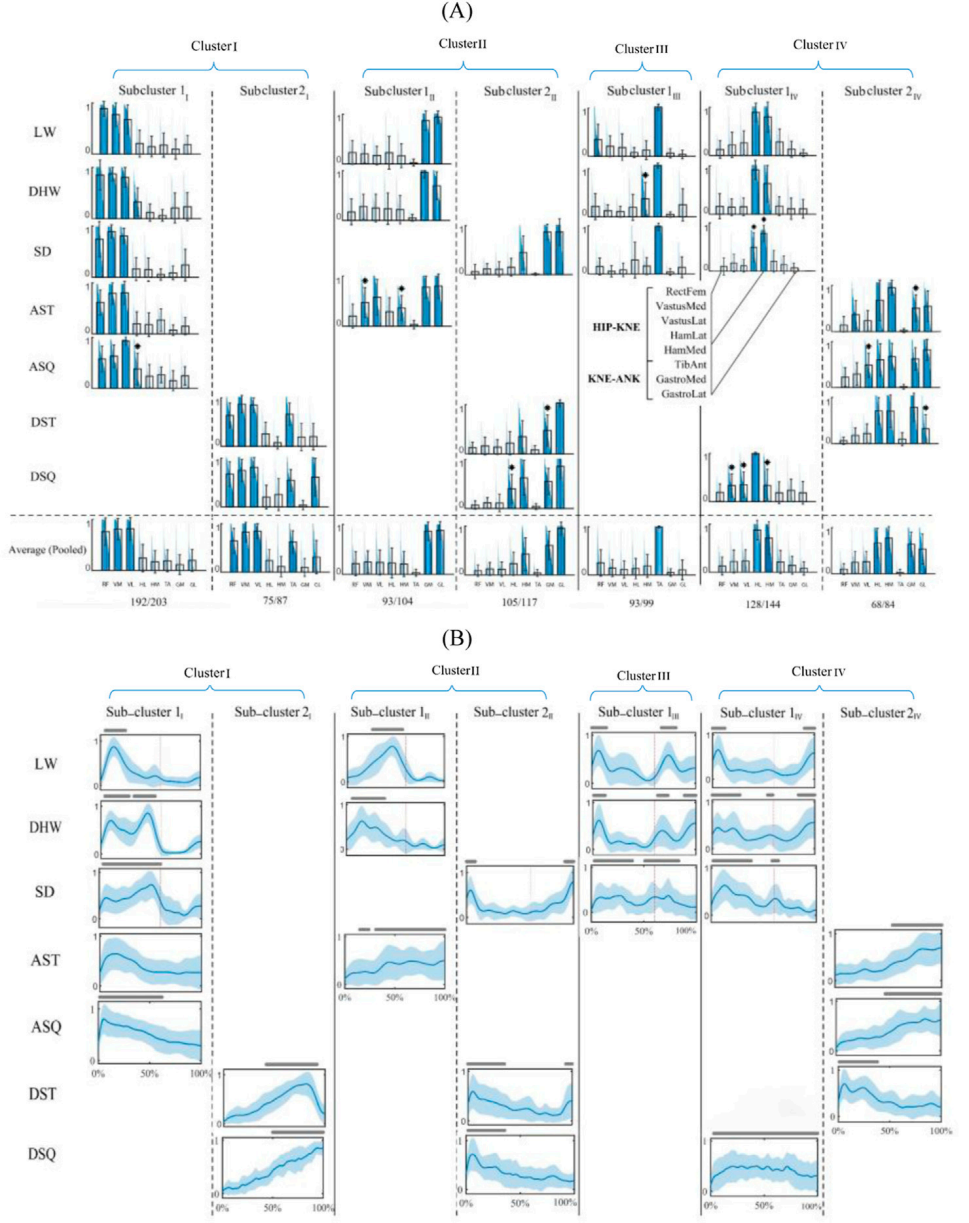
Seven-subcluster arrangement of shared motor modules. **(A)** Characteristic motor modules of different tasks (first seven rows) and the representative motor modules of each subcluster and their coverage rate across all task-trial data (last row). The main contributing muscles into each module (relative weights larger than 0.3) are colored and * sign indicates a significantly different muscle weight from that of the representative module (p < 0.05). **(B)** Activation profiles of the muscle modules. The lines above each profile indicate activation levels above 50% of the maximum and red lines separate the stance and swing phases of the gait cycle. WLV: level walking; WDH: downhill walking; WSD: stair descent, ASQ: squat to stand; AST: sit to stand; DSQ: stand to squat; DST: stand to sit.

The final intra-cluster clustering results indicated a seven-subcluster arrangement for the shared modules (Figure 3A). In this study, subclusters within each cluster are indicated by numbers with Roman numeral subscripts (e.g., 1_I_, 2_I_ for Cluster I), denoting that these subclusters originate from the clustering of Cluster I. Similarly, subclusters 1_II_, 2_II_ belong to cluster II, and so on. This notation ensures clear differentiation between clusters and their respective subclusters. The cluster I was divided into two subclusters 1_I_ and 2_I_, where the first was common in walking-based and in-place ascending tasks (CI: 94%), and the second across in-place descending tasks only (CI: 86%). A similar observation was made for cluster II, with subcluster 1_II_ being shared by the WLV, WDH and AST tasks (CI: 89%), and subcluster 2_II_ by the WSD and in-place descending tasks (CI: 90%). While cluster III remained unchanged as subcluster 1_III_, cluster IV was divided to two subclusters 1_IV_ and 2_IV_, where the first was common across the walking-based and DSQ tasks (CI: 89%), and the second among the in-place ascending tasks and the DST (CI: 81%). The activation profiles of the shared motor modules (Figure 3B) exhibited different temporal patterns, in accordance with the muscle activation data of each specific task.

**FIGURE 4:**
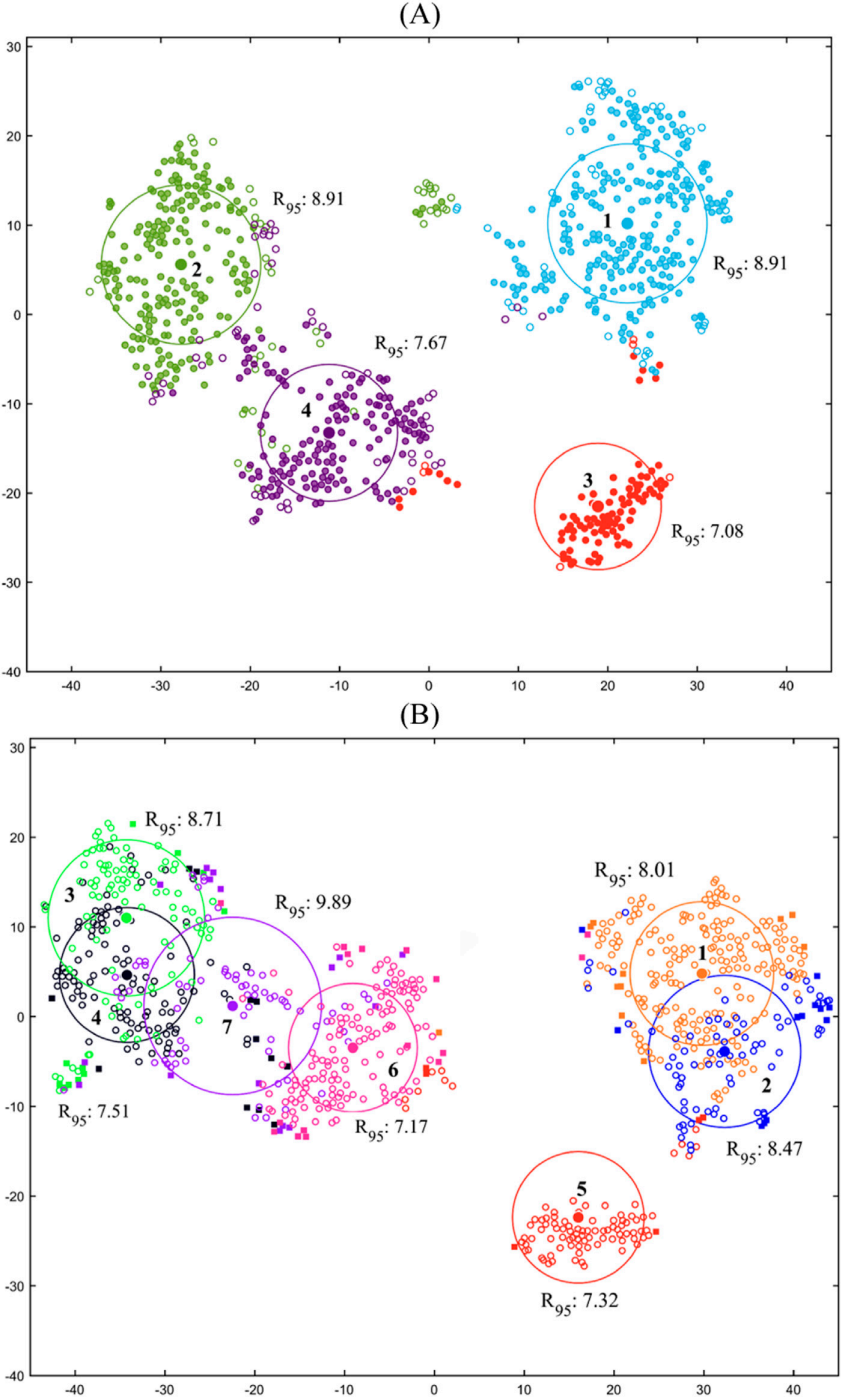
t-SNE plots for the **(A)** four cluster and **(B)** seven-subcluster arrangements of the shared motor modules. The center of each circle represents the centroid of a cluster/subcluster and hollow and solid circles illustrate preserved and eliminated data, respectively.

The t-SNE plots of the four-cluster and seven-subcluster arrangements of the shared motor modules are illustrated in Figure 4. The four-cluster arrangement (Figure 4A) involved relatively good consistency (*R*_*95*_ between 7.08 and 8.91) and distinctness (well-separated *R*_*95*_ circles) based on visual evaluation of the circles but missed more than 16% of the task-trial data. The seven-subcluster arrangement (Figure 4B), resulted in a decrease in the consistency (*R*_*95*_ between 7.17 and 9.89) and particularly the distinctness (overlapping *R*_*95*_ circles). However, it improved the coverage index of the task-trial data to about 90%.

## 4 Discussion

More detailed research on the similarity of muscle synergies across different tasks helps to obtain a deeper insight into the organization of motor modules by the central nervous system to overcome the motor control complexities. This study investigated the generalizability of the motor modules across two families of walking-based and in-place motor tasks, in TKR individuals with similar ages. In particular, this study employed a more sophisticated methodology, based on multi-level k-means clustering, which enabled identifying the representatives of the shared motor modules, considering the whole task-trial data, and assessing their representability quantitatively.

The results of this study for the number of muscle synergies (Figure 1) indicated a significantly larger number of modules for the walking-based tasks in comparison with the in-place tasks (*p* < 0.05), which is a reflection of their higher motor complexity. Also, among the walking-based tasks, the WSD and WDH had larger number of synergies than the WLV (*p* < 0.05); this result might be explained by the fact the WSD and WDH require more antagonistic muscle actions than the WLV to stabilize the body and hence involve a higher degree of motor control complexity. A similar reasoning might be used to explain the high inter-trial variability of the synergy numbers of in-place descending tasks compared to the walking-based and in-place ascending tasks. Unlike the latter tasks which are muscle-driven, the first tasks are mainly accomplished by antagonistic muscle actions to control a potentially variable weight induced motion.

The characteristic muscle synergies found in this study for level walking of TKR patients (Figure 2) are similar to those reported in the literature for healthy population (Neptune et al., 2009; Clark et al., 2010). This result might suggest that despite missing the proprioceptive information provided by cruciate ligaments in intact knees (Johansson et al., 1990; Halata et al., 1999), the motor patterns of TKR patients remain unchanged. Similar results were observed in comparison with those of the healthy population during other tasks, including WDH (Dewolf et al., 2020), WSD (Smale et al., 2016), and in place tasks (Smale et al., 2016; Hanawa et al., 2017).

The inter-task clustering results (Figure 2) indicated a high generalizability of motor modules across different tasks. Four representative common motor modules were identified, with relatively good consistency, distinctness (Figure 4) and representability. In particular, the modules of clusters I and II were common in all walking-based and in-place tasks, with a CI of 83% at least. Also, the modules of cluster III and IV were shared by walking-based tasks and all tasks except for the ASQ, respectively, with a CIs of 94% and 76%. Nevertheless, for some tasks, significant differences were observed in the detailed structures of the characteristic modules compared to the representative shared modules, e.g., ASQ and DSQ in cluster I (Figure 2), which might suggest that the criteria employed for the generalizability analysis were insufficient.

In an attempt to impose more restrictive criteria on the generalizability analysis and identify representative shared motor modules with higher CIs, each of the clusters I, II and IV were divided into two subclusters, providing seven representative shared modules in total (Figure 3). As a consequence, the generalizability of the motor modules was reduced and task-specific modules were emerged for the tasks. More specifically, four representative common motor modules (subclusters 1_I_, 1_II_, 1_III_ and 1_IV_) were identified for all walking-based tasks, except for the WSD which had a single shared module with the in-place descending tasks (subcluster 2_II_). Also, two representative common modules were found for the in-place ascending tasks (subclusters 1_I_ and 2_IV_), where the AST had an additional module in common with the walking-based tasks. Finally, two representative shared modules were identified specifically for in-place descending tasks, with one additional module for each of the DST and DSQ in common with the in-place ascending and the walking-based tasks, respectively. Although the representability of the shared motor modules was improved from 84% to 90% of the task-trial data, their consistency and distinctness were reduced (Figure 4). Based on these observations, it might be suggested that there are shared motor modules across walking-based and in-place tasks in TKR individuals, with their generalizability and representability depending upon the analysis method. With a less restrictive analysis, such as the four-cluster arrangement (Figure 2), the motor modules appear to be highly generalizable, even across tasks of different families. However, with more strict criteria, as that of seven-subcluster arrangement (Figure 3), the generalizability reduces to the tasks of the same family mainly, although there remain some inter-family shared modules. Moreover, in spite of the fact the latter method improves the representability of the shared motor modules, it might cause reduced consistency and distinctness. These findings suggest that more detailed investigations are required to provide a deeper insight into the generalizability of the motor modules across different tasks.

While the clustering results indicated that motor modules could be shared across tasks, the activation profiles revealed notable differences in their recruitment patterns. This suggests that although similar muscle synergies are employed for different tasks, their temporal coordination varies depending on task demands. Such differences in activation patterns highlight the adaptability of neuromuscular control, indicating that motor modules are not rigidly executed but are flexibly modulated to accommodate distinct movement requirements. This finding underscores the importance of analyzing both the structure and timing of muscle synergies when studying motor control strategies across different tasks.

This study introduced a distribution-based approach to address a key limitation of previous motor module generalizability analyses. Prior studies commonly relied on averaged motor modules (Hug et al., 2011; Barroso et al., 2014; Routson et al., 2014), which may obscure important trial-specific variations and inter-subject differences. By considering the full distribution of motor modules across trials rather than a single mean representation, this method ensures that both common and task-specific motor modules are identified more accurately. The advantage of this approach is evident in how the shared modules were distributed across tasks (Figure 3), revealing both highly generalizable synergies and task-specific adaptations.

This study suffers from some limitations that should be addressed in future investigations. First of all, the number of subjects and the muscles under study were limited. A larger number of subjects would make the results statistically more reliable. Also, considering the critical role of motor coordination in the synergy analysis, investigation of a larger number of muscle actions can provide more meaningful synergy data. Moreover, the surface EMG signals are usually subjected to noise and cross-talk. Recording the EMG signals using needle electrodes might provide more trustworthy data. Furthermore, a threshold-based correlation analysis was used as the primary method for identifying common motor modules; other methods might be employed in future studies to assess these findings. Additionally, although these results supported the generalizability of motor modules across different tasks and even task families, it is not clear whether this observation can be generalized across all motor tasks. Future studies shall investigate a wider range of activities to provide more insight into the motor generalizability concept. Moreover, the absence of longitudinal data is another limitation, as motor modules may evolve pre- and post-surgery or through rehabilitation. Tracking changes over time would provide insight into neuromuscular plasticity and recovery trajectories in TKR patients. Finally, it is also important to note that this investigation focused specifically on TKR patients rather than individuals with unicompartmental or combined knee implants (Rossi et al., 2021; Andriollo et al., 2024). The partial knee replacements can exhibit distinct biomechanical characteristics and clinical outcomes compared to TKRs. Therefore, the findings presented here may not be directly generalizable to those with partial knee replacements, and future studies are encouraged to explore whether similar or different motor modules emerge in those populations.

Moreover, future research could explore the integration of these muscle synergy findings with kinematic modularity analyses of walking-based and in-place tasks (Darvishi et al., 2022b) to gain a more comprehensive understanding of the interplay between muscular and kinematic coordination. Such combined analyses may help in tracking changes in both kinematic and muscular modules during rehabilitation, potentially serving as a valuable parameter for personalized treatment. Furthermore, these insights could be incorporated into smartphone-based care management platforms (Rossi et al., 2024) to monitor patients remotely and guide real-time adjustments to therapy plans based on evolving neuromuscular and kinematic patterns.

## 5 Conclusion

The generalizability of motor modules across two families of walking-based and in-place motor tasks was investigated in individuals who have undergone total knee replacement surgery. It was found that there are shared motor modules across tasks, even those of different families, with their generalizability and representability depending upon the analysis method. Less strict criteria result in highly generalizable modules across diverse tasks, while stricter criteria limited generalizability mainly to tasks of the same nature. This finding highlights the importance of the analysis method in identifying the shared motor modules, as the main building blocks of motor control, and provides insight into their organization by the central nervous system to overcome the motor control complexities.

## Data Availability

The original contributions presented in the study are included in the article/supplementary material, further inquiries can be directed to the corresponding author.
